# Plasticity of NMDA receptor NR2B subunit in memory and chronic pain

**DOI:** 10.1186/1756-6606-2-4

**Published:** 2009-02-03

**Authors:** Min Zhuo

**Affiliations:** 1 Department of Physiology, Faculty of Medicine, University of Toronto Centre for the Study of Pain, University of Toronto, 1 King's College Circle, Toronto, Ontario M5S 1A8, Canada

## Abstract

Glutamatergic synapses play critical roles in brain functions and diseases. Long-term potentiation (LTP) is a most effective cellular model for investigating the synaptic changes that underlie learning as well as brain disease – although different molecular mechanisms are likely involved in LTP in physiological and pathological conditions. In the case of learning, N-methyl-D-aspartate (NMDA) receptor is known to be important for triggering learning-related plasticity; alpha-amino-3-hydroxy-5-methyl-4-isoxazolepropionic (AMPA) receptors are thought to be important for the expression of synaptic changes. In this review, I will examine recent evidence on the novel roles of NMDA receptors, in particular NR2B subunit-containing NMDA receptors in learning and chronic pain. A *positive feedback *control of NR2B receptor subunit is proposed to explain cortical sensitization involved in chronic pain, but not learning and memory.

## Introduction

The NMDA receptor acts as an activity-dependent coincidence detector in the central nervous system (CNS). The majority of past research has focused on the synaptic changes – namely presynaptic changes in glutamate release, and postsynaptic AMPA receptor changes – following the activation of the NMDA receptor. Few review articles are available about the potential long-term plastic changes in NMDA receptor subunits, in particular NR2B subunit. In this review, by focusing on physiological (memory) and pathological (chronic pain) functions, I will examine previous and recent evidence for long-term plastic changes of NMDA receptor NR2B subunit in the central sensory and 'learning' synapses, as well as the molecular machinery that may contribute to NMDA receptor NR2B subunit trafficking and postsynaptic insertion.

## LTP as a cellular model for brain plasticity

It is well known that central synapses are highly plastic, and long-term changes in synaptic transmission contribute to different functions of the brain throughout the lifespan. Two major forms of synaptic plasticity have been widely investigated: long-term potentiation (or called LTP) and long-term depression (or called LTD). While LTP can enhance synaptic functions in certain regions of the brain, LTD attenuates or reduces the efficacy of synaptic transmission. Such biphasic synaptic plasticity is not limited at excitatory, glutamatergic synapses. Both LTP and LTD have been also reported in inhibitory synapses, and underlying cellular and molecular mechanisms are different. Recent studies using different induction protocols reveal that the mechanisms for central LTP are likely to be different, depending the induction protocols, regions of the CNS, input fibers and postsynaptic neurons recorded [1-5]. There is no doubt that many different molecular targets will be continuously revealed in future, one urgent task is to verify the physiological or pathological relevant of synaptic LTP/LTD induced by experimental induction protocols. Furthermore, new forms of LTP and LTD remain to be discovered to mimic physiological and/or pathological changes under *in vivo *conditions (e.g., presynaptic enhancement of neurotransmitter release after tissue injury in the anterior cingulate cortex (ACC) (see [6]).

What has been recognized about the potential functions of LTP is its contribution to many key brains functions in addition to learning and memory [7-11]. At the spinal cord dorsal horn where the first sensory synapses are located, LTP of sensory synaptic transmission can be induced by different experimental protocols [12-14] or peripheral injury [14]. Potentiated excitatory synaptic transmission is believed to contribute to spinal sensitization that at least in part attributes to behavioral hyperalgesia and allodynia during chronic pain. In the basolateral amygdala, LTP can be induced between thalamic/cortical inputs and postsynaptic principle neurons [15] or fear conditions [11], such enhanced responses are important for encoding fearful information. In the hippocampal CA1 region where most of LTP studies have been reported, LTP can be induced and reliable detected, even with field recording electrodes [7,8]. However, despite a huge amount of literature on hippocampal LTP, it remains to be demonstrated that if a simple spatial training trial may induce LTP in certain population of CA1 neurons.

Finally, in the prefrontal cortical (PFC) neurons including the ACC, LTP is induced by the pairing, spike-timing and theta burst protocols [16] as well as peripheral injury [17,6]. It has been proposed that the injury caused synaptic potentiation contribute to chronic pain and pain-related high brain functions including fear and emotion [18]. Therefore, it is clear that studying central LTP provides fundamental mechanisms for brain functions – from pain transmission to fear and chronic pain.

## NMDA receptor and LTP

One major feature of LTP is the requirement of activation of NMDA receptors. As compared with downstream signaling protein inhibitors, the inhibition of NMDA receptor by bath application of AP-5 reliably blocks the induction of LTP. In most cases, basal synaptic responses are not affected the same application of AP-5, indicating the selective roles of NMDA receptors in the induction. The key mechanism for the involvement of NMDA receptor in the induction of LTP is its voltage-dependence. At resting membrane potentials, NMDA receptors are inactive due to pore blockade by extracellular Mg^2+^, even in the presence of glutamate. Thus, in order to activate NMDA receptors at synapses, two events need to happen simultaneously. First, glutamate needs to be released and binds to NMDA receptors; second, the postsynaptic membrane needs to be depolarized so that the block by extracellular Mg^2+ ^can be removed. NMDA receptor-mediated calcium influx from the extracellular space into the postsynaptic cells then activates a series of signaling molecules within postsynaptic cells, including protein kinases, protein phosphatases, immediate early genes (i.e., genes can be activated rapidly, or called third messengers), as well as enzymes producing diffusible retrograde messengers. Although the requirement for NMDA receptor in LTP is easily found, application of NMDA did not cause synaptic potentiation [7], making biochemical studies of LTP difficult.

The requirement of the activation of NMDA receptors in synaptic LTP is common among many brain regions. In the spinal cord dorsal horn neurons, LTP of AMPA receptor mediated EPSCs were induced by the induction protocol that paired synaptic stimulation (2 Hz) with postsynaptic depolarization (+30 mV) [12]. In the amygdala, LTP induced by the pairing protocol is blockaded by NMDA receptor antagonist AP-5 [19]. In the ACC, bath application of a NMDA receptor antagonist AP-5 completely abolished the induction of LTP induced by different induction protocols in the pyramidal cells [16], indicting that the induction of ACC LTP is completely dependent of postsynaptic activation of NMDA receptors.

## NMDA receptor independent LTP

NMDA receptor independent LTP have been also reported. Most of these experiments showing NMDA receptor independent LTP are performed in the presence of a NMDA receptor blocker. It has to be noted that some experiments only used a sub-dose of AP-5. It is critical to demonstrate by whole-cell patch-clamp recording that the same dosage of NMDA receptor antagonist does in fact completely eliminate NMDA receptor mediated excitatory postsynaptic currents (EPSCs) [16]. Depending on the regions of the CNS and the stimulation protocols, there are many reports of NMDA receptor as an independent form of LTP. Several neurotransmitter receptors or ion channels have been implicated in the initiation of LTP, such as L-type voltage gated calcium channels (L-VGCCs), metabotropic glutamate receptors (mGluRs), serotonin receptors, dopamine receptors and kainate (KA) receptors. For example, in the hippocampus, in the presence of AP-5, very strong tetanic stimulation or bath application of TEA induced LTP that is sensitive to the blockade of L-VGCCs [20,21]. Bath application of tACPD, an agonist of mGluRs, produced long-lasting potentiation that may require cGMP-related signaling pathways [22]. In a recent study using gene knockout of KA receptor subtype 6 (KA GluR6) mice, the pairing induced LTP was reduced or blocked in the ACC and amygdala, suggesting that KA receptor may contribute to synaptic potentiation [23].

## Composition of NMDA receptors

Functional NMDA receptors contain heteromeric combinations of the NR1 subunit plus one or more of NR2A-D. While NR1 distributes ubiquitously in the CNS, NR2 subunits exhibit regional distribution, and the amount of expression is developmentally related. In the neonatal brains, NR2B and NR2D subunits are highly expressed, and over the course of development, they are substituted or replaced by NR2A and NR2C. In humans and rodents, NR2A and NR2B subunits predominate in forebrain structures [24,25]. NR2A and NR2B subunits confer distinct properties to NMDA receptors; heteromers containing NR1 plus NR2B mediate a current that decays three to four times more slowly than receptors composed of NR1 plus NR2A [25-27]. Unlike other ionotropic channels, NMDA receptors are 5–10 times more permeable to calcium, a critical intracellular signaling molecule for triggering postsynaptic and possible presynaptic plastic changes, than to Na^+ ^or K^+^. NMDA receptor mediated currents are long-lasting compared with the rapidly desensitizing kinetics of AMPA and KA receptor channels.

## Contribution of NR2B-containing NMDA receptors to synaptic LTP

**Figure 1:**
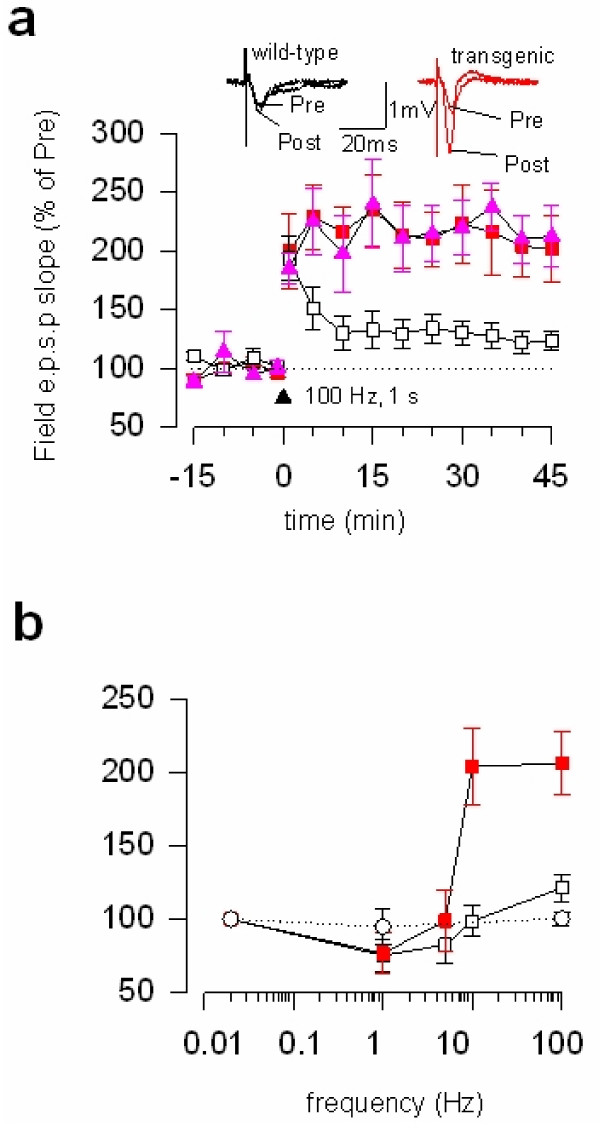
**Genetic overexpression of NMDA NR2B receptors in forebrains enhanced central synaptic potentiation**. **a**. Synaptic potentiation induced by a single tetanic stimulation (100 Hz, 1 sec) was significantly enhanced in NR2B transgenic mice compared with wild-type littermates; **b**. Enhancement of synaptic potentiation is frequency-dependent, and LTD induced by low-frequency stimulation (1 Hz, 15 min) was not affected din NR2B transgenic mice. Modified from Tang et al (1999). Zhuo's lab contributed to the plasticity studies in this original article.

The requirement of NR2B-NMDA receptors in synaptic potentiation has been reported in different areas of the CNS (see Table 1). Considering the NMDA receptor mediated currents are largely carried out by NR2A-containing NMDA receptors, one expects that inhibition of NR2B receptor alone may not be sufficient to produce complete blockade of synaptic potentiation. Indeed, in the ACC synapses, bath application of selective NMDA receptorNR2B antagonists significantly reduced but not blocked the induction of LTP by the pairing protocol [16]. Similarly, in the lateral amygdala, NMDA receptor NR2B antagonist Ro 25–6981 significantly reduced NMDA receptor-dependent LTP induced by a pairing protocol [28]. In the hippocampal CA1 region, some studies have reported that the activity of NR2B-NMDARs is not required for synaptic potentiation or LTP [29]. However, a previous study using genetic overexpression of NR2B subunits indicate the involvement of NMDA NR2B receptor. In the hippocampus of transgenic mice with NMDA NR2B overexpression, LTP induced by tetanic stimulation or repetitive stimulation were significantly enhanced as compared with wild-type littermates [30] (Figure 1).

**Table 1:** Summary of the involvement of NMDA receptor NR2B subunit in synaptic LTP

***Brain region***	***Pharmacological inhibitors or genetic manipulation***	***Induction protocol***	***Effects on LTP***	***Reference***
**ACC**	NR2B antagonist	Pairing protocol	Reduced	Zhao et al., 2005
		Spike-timing protocol	Blocked	
		Theta burst stimulation (TBS)	blocked	

**Perirhinal cortex**	NR2B antagonist	High frequency stimulation (HFS)	No effect	Massey et al., 2004

**Visual cortex**	NR2B antagonist	Pairing protocol	blocked	Yoshimura et al., 2003

**Hippocampal CA1**	NR2B overexpression	HFS or repetitive stimulation	Enhanced	Tang et al., 1999
		HFS	No effect	Liu et al., 2004
	NR2B antagonist	Pairing protocol	No effect	
		HFS	Reduced	Berberich et al., 2007
		Pairing protocol	Blocked	
		HFS	No effect	Zhang et al., 2008
		Pairing protocol	No effect	
		Spike-timing protocol	blocked	

**Amygdala**	NR2B antagonist	Pairing protocol	Reduced	Miwa et al., 2008

**Spinal dorsal horn**	ND	ND	ND	

Considering that NR2B- and NR2A- NMDA receptors have different biophysical properties and couple to different intracellular signaling cascades, it may be possible that different induction protocols may activate different NMDA receptor subtypes. It has been reported that different LTP-inducing protocols recruit different signaling pathways. For example, in the amygdala, pairing-protocol induced LTP depends on L-VGCCs but not NMDA receptors, while tetanus-stimulation induced LTP involves NMDA receptor but not L-VGCCs [31]. Recently, we have shown that the involvement of NR2B-NMDA receptors in hippocampal LTP are dependent on induction protocols [32,33]. NR2B-NMDA receptors are required for LTP induced by the spike-timing protocol, but not by the pairing protocol [5]. The same dose of Ro 25–6981 reduced LTP induced by the spike-timing protocol, but not by the pairing protocol. Interestingly, neither LTP induced by two train tetanic stimulation nor late-phase LTP induced by multiple train stimulation was affected by NR2B antagonist Ro 25–6981. Furthermore, calcium imaging studies showed that the NR2B-NMDA receptor mediated Ca^2+ ^transients were faster under the spike-timing than pairing protocols, which might explain the different significance of NR2B-NMDARs in LTP under the two protocols since fast Ca^2+ ^transients are better for LTP, but slow Ca^2+ ^transients not [5].

## Requirement of NMDA receptor NR2B subunit in behavioral learning

Although the NMDA NR2B receptor antagonists have been available for a few years, there are a few studies that have investigated the contribution of NMDA NR2B receptors to behavioral learning. For spatial water maze memory, it has been reported that 60-min pretreatment with (+/-)-CP-101,606 (60 mg/kg, p.o.), a dose that fully occupied hippocampal NR1/NR2B subunit-containing receptors, as determined by ex vivo NMDA receptor-specific ^[3H]^ifenprodil binding immediately following water maze experiments, had no effect on acquisition or the probe trial. These results suggest that antagonists selective for NR1/NR2B subunit-containing receptors may not impair spatial memory in rats in the Morris water maze [34].

Unlike spatial memory, the contribution of NMDA NR2B receptors to fear memory has been reported. Systemic injections of NMDA NR2B receptor antagonist ifenprodil before training led to a dose-dependent impairment in the acquisition of auditory and contextual fear conditioning, whereas injections before testing had no effect [35]. Recently, similar administration of NMDA receptor NE2B subunit antagonist Ro 25–6981 significantly attenuated fear extinction but not re-extinction recall [36]. In the hippocampal CA1 region, pre-training intra-CA1 infusion of ifenprodil or Ro 25–6981 impaired the contextual fear memory induced by five CS-US pairings, with no effect on the memory induced by one conditioned stimulus (CS)-unconditioned stimulus (US) pairing [5]. These findings are in good accord with previous genetic studies that forebrain NR2B overexpression enhanced spatial and fear memory [30]. Furthermore, intra-amygdala infusions of ifenprodil mirrored systemic injection results and, in addition, showed that the effects are attributable to a disruption of fear learning rather than a disruption of memory consolidation. NMDA receptors in lateral amygdala are thus involved in fear conditioning, and the NR2B subunit appears to make unique contributions to the underlying plasticity [35]. In addition to the amygdala, a recent study indicates that neurons in the ACC may also contribute to the formation of fear memory. Inhibition of NMDA NR2B functions in the ACC by local microinjection of pharmacological NR2B antagonists or NR2B siRNA manipulation significantly reduced fear memory [16], suggesting that cortical NMDA receptor NR2B subunit also contribute to fear memory formation.

## NMDA NR2B receptor does not undergo potentiation in memory storage

Unlike AMPA receptors during early phase memory, it is generally believed that NMDA receptors do not undergo rapid and prolonged changes during memory (or hippocampal LTP) (Table 2). Systemic administration of NMDA NR2B antagonists after the training and before the testing did not affect fear memory [35]. Consistent with this finding, AMPA receptor mediated responses are found to be enhanced after fear conditioning in the amygdala and NMDA-mediated transmission in the thalamic-to-lateral amygdala pathway is not facilitated after fear conditioning. Western blots show a reduction in phosphorylated-NR1, NR2A, and NR2B subunit protein expression in the amygdala from fear-conditioned animals. There are at least three possible physiological significances for the reduction in NMDA receptor NR2B functions [37]. First, the down-regulation of the NMDA receptor may protect against neuronal excitotoxicity of unchecked NMDA receptor recruitment during the induction and consolidation of fear memories. It is well known that NMDA NR2B receptor antagonists have neuroprotective effects. Second, reduced NMDA current and protein may allow persistence of the "capacity to reactivate" amygdala pathways for the induction of future fear memories. The overexcitation caused by the upregulation of NMDA receptors may prevent the formation of new fear memory that is critical for animals to survive in the natural environment. Finally, a persistent long-term depression of NMDA transmission may occur after fear learning [37]. Similar results have been found in the visual cortex. Experience-dependent plasticity is reported to cause changes in the NR2A:NR2B ratio, favoring the expression of NMDA receptor NR2A subunit [38-41].

**Table 2:** Summary of the involvement of NMDA receptor NR2B subunit in behavioral memory and chronic pain

***Brain function***	***Drug or manipulation***	***Brain regions***	***Effects***	***Reference***
**Spatial memory (Morris water maze)**	NR2B overexpression	Forebrain	Enhanced fear memory	Tang et al., 1999

	NR2B antagonist	Systemic injection	No effect	Guscott et al., 2003

**Fear memory**	NR2B overexpression	Forebrain	Enhanced fear memory	Tang et al., 1999
	NR2B antagonist	Amygdala	Blocked acquisition	Rodrigues et al., 2001
			No effect on expression of fear	
	NR2B antagonist	Amygdala	Inhibit fear conditioning without effect on fear expression	Walker and Davis, 2008

**Chronic pain**	NR2B overexpression	Forebrain	Enhancing chronic pain	Wei et al., 2002
	NR2B antagonist	ACC	Inhibiting chronic pain	Wu et al., 2005

## Upregulation of NMDA receptor NR2B subunit functions in chronic pain

**Figure 2:**
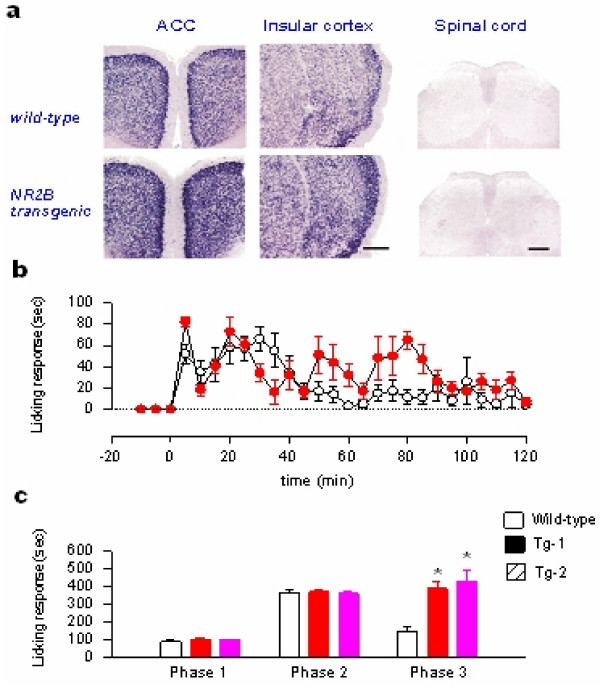
**Selective enhancement of chronic pain in 'smart' mice with NR2B overexpression in forebrains**. **a**. Overexpression of NR2B mRNA in pain-related forebrain areas including the ACC and insular cortex (IC). No overexpression of NR2B was detected in the spinal cord. **b**. Enhancement of behavioral nociceptive licking responses to peripheral subcutaneous injection of formalin in NR2B transgenic mice. **c**. Summarized three different phases of behavioral nociceptive licking responses in wild-type and NR2B transgenic mice. Similar increases in behavioral nociceptive responses were found in the second line of NR2B transgenic mice. Modified from Wei et al. (2001).

**Figure 3:**
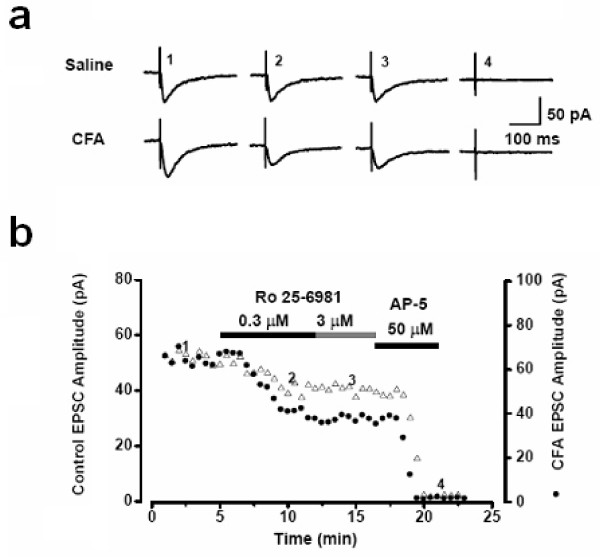
**Peripheral inflammation triggers long-term increases in NMDA NR2B receptor mediated currents in cingulate pyramidal cells**. NR2B sensitive component of NMDA receptor mediated EPSCs were enhanced in mice with CFA injection. **a**. Traces of EPSCs show the currents at different time points during application of drugs. Ro 25–6981 produced its maximal effect at 3 min after bath application, and a higher dose of Ro 25–6981 (3 μM) had no additional effects. The remaining currents can be totally blocked by AP-5 (50 μM). **b**. A selective NR2B antagonist, Ro 25–6981, partially inhibited NMDA receptor-mediated EPSCs. The time course of changes in EPSC amplitude before and during the application of Ro 25–6981 (0.3 and 3 μM) and AP-5 (50 μM) in ACC neurons from both saline (control, open symbols) and CFA-injected (filled sumbols) mice is shown. Modified from Wu et al. (2005).

In NMDA receptor NR2B subunit genetically overexpression mice, we found that chronic pain – but not acute or physiological pain – was selectively enhanced [30,42] (see Figure 2), providing the first genetic evidence that forebrain NMDA NR2B receptor is critical for chronic pain. This finding also offers additional reasons to explain why NMDA NR2B receptor is not undergoing upregulation during learning (see above). Does genetically overexpression of NR2B mimic physiological or pathological conditions? In a recent study we found that after persistent inflammation (the Complete Freund's Adjuvant (CFA) animal model for chronic inflammation), the expression of NMDA NR2B receptors in the ACC was increased over a long-period of time, thereby increasing the NR2B component in NMDA receptor mediated EPSCs [17] (see Figure 3A, B). In the behavioral allodynia test, microinjection into the ACC or systemic administration of NMDA NR2B receptor with selective antagonists inhibited behavioral responses to peripheral inflammation [17]. These results are consistent with genetic studies that mice with NMDA receptor NR2B subunit forebrain overexpression selectively enhanced inflammation-related persistent pain without significant changes in acute pain [43]. The anti-allodynic effects of NMDA NR2B receptor antagonists have been also reported in other animal models of chronic pain. We believe that these findings provide direct evidence that NMDA NR2B receptors undergo long-term plastic changes in the brain after injury.

**Figure 4:**
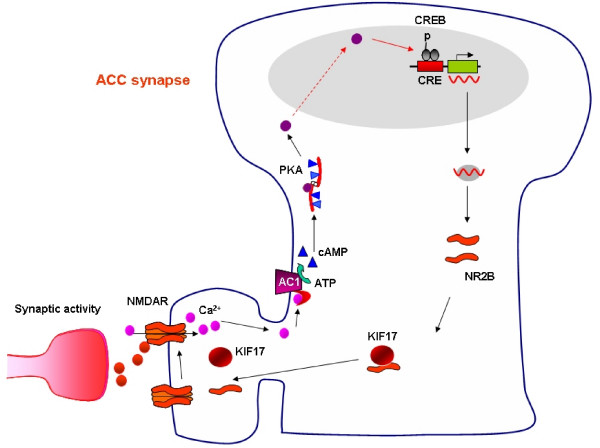
**Central signaling pathways contribute to chronic pain**. In the ACC, glutamate is the major fast excitatory transmitter between input fibers and pyramidal cells. Peripheral injury such as tissue inflammation or nerve injury trigger a burst of abnormal activity in the ACC circuits; and subsequently activate postsynaptic NMDA receptors on cingulate pyramidal cells located in layer II-III. Activation of NMDA receptor triggers calcium influx. In adult ACC pyramidal cells, most of NMDA receptors are the combination of NR1-NR2A, NR1-NR2B with possible minor component of currents made of NR1-NR2A-NR2B. Postsynaptic increases in Ca^2+ ^leads to activation of Ca^2+^-calmodulin (CaM) dependent pathways. Among them, Ca^2+ ^and CaM stimulated AC1 is activated, and this activation leads to the generation of the key second messenger cAMP. Subsequently, cAMP activates PKA. PKA then translocates to the nucleus and phosphorylates CREB. NR2B contains a CREB binding domain which may couple increases in intracellular calcium with the increase in NR2B expression. Subsequently, postsynaptic synthesis of NMDA NR2B is increased, and together with endogenous motor protein KIF17, these new NR2B subunits are added to postsynaptic NMDA receptors. Such *positive feedback control *may further enhance neuronal excitability within the ACC, and contribute to chronic pain.

Recent studies in other central synapses suggest that NMDA NR2B receptor up-regulation is likely to be reliant on activity-dependent mechanisms. The molecular motor protein KIF 17 has been shown to be involved in the active transport of NMDA receptor NR2B subunits [44-46]. NR2B contains a cAMP response element-binding protein (CREB) binding domain which may couple increases in intracellular calcium with the increase in NR2B expression. Since NMDA receptors play an important role in activity-dependent plasticity in the ACC, we suggest that NMDA NR2B subunit may be regulated through NMDA-calcium-CaM-dependent signaling pathways. The activation of NMDA receptors triggers postsynaptic calcium, leading to the activation of calcium-stimulated CREB in the ACC after peripheral or central injury [42] (see Figure 4).

## cAMP as a key second messenger

Studies using gene knockout of calcium-calmodulin dependent adenylyl cycles AC1 and AC8 revealed that AC1 pays key roles in triggering injury related central plasticity. In AC1 knockout mice, LTP, a likely synaptic model for chronic pain in the cortex, was completely abolished [47]. The effects of inhibition of AC1 are selective as both basal synaptic responses (mostly mediated by AMPA receptor) and NMDA receptor mediated responses were not affected [47,18]. Because of the ACC LTP induced by the paring protocol is mediated by postsynaptic AMPA receptor (likely through postsynaptic receptor trafficking) [48], it is suggested that AC1 activity is critical for calcium-triggered AMPA receptor trafficking. Consistent with in vitro slice findings, behavioral responses in different animal models of chronic pain are significantly reduced or blocked in AC1 knockout mice.

Recent studies using the animal model of neuropathic pain actually show that postsynaptic AMPA receptor mediated responses are enhanced in the ACC synapses of mice receiving nerve injury. The enhancement of AMPA receptor mediated EPSCs is likely through the phosphorylation of postsynaptic AMPA receptor GluR1 subunit at PKA site [6]. Such nerve injury triggered enhancement of postsynaptic responses and GluR1 receptor phosphorylation are abolished in AC1 knockout mice, suggesting that AC1 dependent-cAMP pathway is important. In addition to postsynaptic mediated effects, presynaptic enhancement of glutamate releases in the ACC synapses have been also found, and AC1 is also required for triggered these presynaptic changes [6]. It remains to be investigated if such AC1 contribution is directly on presynaptic terminals, or act through possible retrograde messengers in the ACC [18].

Studies using AC1 knockout mice also demonstrated that AC1 activity is critical for NR2B upregulation after the injury. Changes in NMDA NR2B antagonist sensitive EPSCs caused by the nerve injury were abolished in mice lacking AC1 (unpublished data). It is possible that NMDA NR2B receptor-AC1-cAMP-CREB-NR2B form a positive cellular feedback to reinforce the NMDA receptor functions in the ACC neurons. Together with NMDA receptor mediated AMPA receptor potentiation, these synaptic changes greatly enhance excitatory synaptic transmission in the ACC after the injury, and thus contribute to cortical pain (see Figure 4).

## Clinical considerations for developing NR2B agonists as memory enhancers or antagonists as pain killers

Based on the discovery of NMDA NR2B receptor functions in LTP and behavioral memory, there are several proposals of developing NMDA NR2B function enhancer to increase memory functions in so-called low IQ adults or to rescue the memory loss in the patients. This hypothesis gains some supports from the experiments of the environment enrichment in that both NMDA NR2B functions and behavioral learning are significantly enhanced. However, considering the negative finding of NMDA NR2B receptors in the expression of memory, it is unlikely that enhancing NMDA NR2B functions will be beneficial for maintaining newly formed memory (see above). Furthermore, enhancing NMDA NR2B functions may enhance chronic pain in case of any tissue or nerve injury [18]. In addition to chronic pain, the potential roles of NMDA NR2B receptors in cell death, seizure have been reported. Enhancing NR2B receptors may also enhance these potential risks.

To use the NMDA receptor NR2B antagonists to control brain disease and chronic pain have been reported by many groups. For example, in the case of chronic pain, it has been reported that NR2B antagonists produced powerful analgesic effects in animal models of chronic pain (see above). The action off site for NR2B antagonist is mainly due to cortical NR2B receptors, although it may also exert some of its effects in the spinal cord level. Alternatively, in the case that side effects in humans are found with NR2B antagonists, one may target on downstream proteins such as AC1. In summary, we are just beginning to understand how central synapses may undergo plastic changes during learning or after peripheral tissue injury, understanding basic mechanisms for such long-term plasticity may help us to design better medicines for treating memory loss and chronic pain.

## Competing interests

The authors declare that they have no competing interests.

## References

[B1] Clayton DA, Mesches MH, Alvarez E, Bickford PC, Browning MD (2002). A hippocampal NR2B deficit can mimic age-related changes in long-term potentiation and spatial learning in the Fischer 344 rat. J Neurosci.

[B2] Le Roux N, Amar M, Moreau A, Fossier P (2007). Involvement of NR2A- or NR2B-containing N-methyl-D-aspartate receptors in the potentiation of cortical layer 5 pyramidal neurone inputs depends on the developmental stage. Eur J Neurosci.

[B3] Yoshimura Y, Ohmura T, Komatsu Y (2003). Two forms of synaptic plasticity with distinct dependence on age, experience, and NMDA receptor subtype in rat visual cortex. J Neurosci.

[B4] Hoffman DA, Sprengel R, Sakmann B (2002). Molecular dissection of hippocampal theta-burst pairing potentiation. Proc Natl Acad Sci USA.

[B5] Zhang XH, Liu F, Chen Q, Zhang CL, Zhuo M, Xiong ZQ, Li BM (2008). Conditioning-strength dependent involvement of NMDA NR2B subtype receptor in the basolateral nucleus of amygdala in acquisition of auditory fear memory. Neuropharmacology.

[B6] Xu H, Wu LJ, Wang H, Zhang X, Vadakkan KI, Kim SS, Steenland HW, Zhuo M (2008). Presynaptic and postsynaptic amplifications of neuropathic pain in the anterior cingulate cortex. J Neurosci.

[B7] Bliss TV, Collingridge GL (1993). A synaptic model of memory: long-term potentiation in the hippocampus. Nature.

[B8] Malenka RC, Nicoll RA (1999). Long-term potentiation–a decade of progress?. Science.

[B9] Kandel ER (2001). The molecular biology of memory storage: a dialogue between genes and synapses. Science.

[B10] Malinow R, Malenka RC (2002). AMPA receptor trafficking and synaptic plasticity. Annu Rev Neurosci.

[B11] Rodrigues SM, Schafe GE, LeDoux JE (2004). Molecular mechanisms underlying emotional learning and memory in the lateral amygdala. Neuron.

[B12] Wei F, Vadakkan KI, Toyoda H, Wu LJ, Zhao MG, Xu H, Shum FW, Jia YH, Zhuo M (2006). Calcium calmodulin-stimulated adenylyl cyclases contribute to activation of extracellular signal-regulated kinase in spinal dorsal horn neurons in adult rats and mice. J Neurosci.

[B13] Ikeda H, Heinke B, Ruscheweyh R, Sandkuhler J (2003). Synaptic plasticity in spinal lamina I projection neurons that mediate hyperalgesia. Science.

[B14] Ikeda H, Stark J, Fischer H, Wagner M, Drdla R, Jager T, Sandkuhler J (2006). Synaptic amplifier of inflammatory pain in the spinal dorsal horn. Science.

[B15] Tsvetkov E, Shin RM, Bolshakov VY (2004). Glutamate uptake determines pathway specificity of long-term potentiation in the neural circuitry of fear conditioning. Neuron.

[B16] Zhao MG, Toyoda H, Lee YS, Wu LJ, Ko SW, Zhang XH, Jia Y, Shum F, Xu H, Li BM, Kaang BK, Zhuo M (2005). Roles of NMDA NR2B subtype receptor in prefrontal long-term potentiation and contextual fear memory. Neuron.

[B17] Wu LJ, Toyoda H, Zhao MG, Lee YS, Tang J, Ko SW, Jia YH, Shum FW, Zerbinatti CV, Bu G, Wei F, Xu TL, Muglia LJ, Chen ZF, Auberson YP, Kaang BK, Zhuo M (2005). Upregulation of forebrain NMDA NR2B receptors contributes to behavioral sensitization after inflammation. J Neurosci.

[B18] Zhuo M (2008). Cortical excitation and chronic pain. Trends Neurosci.

[B19] Sigurdsson T, Doyere V, Cain CK, LeDoux JE (2007). Long-term potentiation in the amygdala: a cellular mechanism of fear learning and memory. Neuropharmacology.

[B20] Grover LM, Teyler TJ (1990). Two components of long-term potentiation induced by different patterns of afferent activation. Nature.

[B21] Huang YY, Malenka RC (1993). Examination of TEA-induced synaptic enhancement in area CA1 of the hippocampus: the role of voltage-dependent Ca2+ channels in the induction of LTP. J Neurosci.

[B22] Zhuo M, Laitinen JT, Li XC, Hawkins RD (1999). On the respective roles of nitric oxide and carbon monoxide in long-term potentiation in the hippocampus. Learn Mem.

[B23] Ko S, Zhao MG, Toyoda H, Qiu CS, Zhuo M (2005). Altered behavioral responses to noxious stimuli and fear in glutamate receptor 5 (GluR5)- or GluR6-deficient mice. J Neurosci.

[B24] Dingledine R, Borges K, Bowie D, Traynelis SF (1999). The glutamate receptor ion channels. Pharmacol Rev.

[B25] Cull-Candy S, Brickley S, Farrant M (2001). NMDA receptor subunits: diversity, development and disease. Curr Opin Neurobiol.

[B26] Vicini S, Wang JF, Li JH, Zhu WJ, Wang YH, Luo JH, Wolfe BB, Grayson DR (1998). Functional and pharmacological differences between recombinant N-methyl-D-aspartate receptors. J Neurophysiol.

[B27] Erreger K, Geballe MT, Dravid SM, Snyder JP, Wyllie DJ, Traynelis SF (2005). Mechanism of partial agonism at NMDA receptors for a conformationally restricted glutamate analog. J Neurosci.

[B28] Miwa H, Fukaya M, Watabe AM, Watanabe M, Manabe T (2008). Functional contributions of synaptically localized NR2B subunits of the NMDA receptor to synaptic transmission and long-term potentiation in the adult mouse CNS. J Physiol.

[B29] Liu L, Wong TP, Pozza MF, Lingenhoehl K, Wang Y, Sheng M, Auberson YP, Wang YT (2004). Role of NMDA receptor subtypes in governing the direction of hippocampal synaptic plasticity. Science.

[B30] Tang YP, Shimizu E, Dube GR, Rampon C, Kerchner GA, Zhuo M, Liu G, Tsien JZ (1999). Genetic enhancement of learning and memory in mice. Nature.

[B31] Bauer EP, Schafe GE, LeDoux JE (2002). NMDA receptors and L-type voltage-gated calcium channels contribute to long-term potentiation and different components of fear memory formation in the lateral amygdala. J Neurosci.

[B32] Massey PV, Johnson BE, Moult PR, Auberson YP, Brown MW, Molnar E, Collingridge GL, Bashir ZI (2004). Differential roles of NR2A and NR2B-containing NMDA receptors in cortical long-term potentiation and long-term depression. J Neurosci.

[B33] Berberich S, Jensen V, Hvalby O, Seeburg PH, Kohr G (2007). The role of NMDAR subtypes and charge transfer during hippocampal LTP induction. Neuropharmacology.

[B34] Guscott MR, Clarke HF, Murray F, Grimwood S, Bristow LJ, Hutson PH (2003). The effect of (+/-)-CP-101,606, an NMDA receptor NR2B subunit selective antagonist, in the Morris watermaze. Eur J Pharmacol.

[B35] Rodrigues SM, Schafe GE, LeDoux JE (2001). Intra-amygdala blockade of the NR2B subunit of the NMDA receptor disrupts the acquisition but not the expression of fear conditioning. J Neurosci.

[B36] Dalton GL, Wang YT, Floresco SB, Phillips AG (2008). Disruption of AMPA receptor endocytosis impairs the extinction, but not acquisition of learned fear. Neuropsychopharmacology.

[B37] Zinebi F, Xie J, Liu J, Russell RT, Gallagher JP, McKernan MG, Shinnick-Gallagher P (2003). NMDA currents and receptor protein are downregulated in the amygdala during maintenance of fear memory. J Neurosci.

[B38] Philpot BD, Sekhar AK, Shouval HZ, Bear MF (2001). Visual experience and deprivation bidirectionally modify the composition and function of NMDA receptors in visual cortex. Neuron.

[B39] Sawtell NB, Frenkel MY, Philpot BD, Nakazawa K, Tonegawa S, Bear MF (2003). NMDA receptor-dependent ocular dominance plasticity in adult visual cortex. Neuron.

[B40] Tongiorgi E, Ferrero F, Cattaneo A, Domenici L (2003). Dark-rearing decreases NR2A N-methyl-D-aspartate receptor subunit in all visual cortical layers. Neuroscience.

[B41] Walker DL, Davis M (2008). Amygdala infusions of an NR2B-selective or an NR2A-preferring NMDA receptor antagonist differentially influence fear conditioning and expression in the fear-potentiated startle test. Learn Mem.

[B42] Wei F, Qiu CS, Kim SJ, Muglia L, Maas JW, Pineda VV, Xu HM, Chen ZF, Storm DR, Muglia LJ, Zhuo M (2002). Genetic elimination of behavioral sensitization in mice lacking calmodulin-stimulated adenylyl cyclases. Neuron.

[B43] Wei F, Wang GD, Kerchner GA, Kim SJ, Xu HM, Chen ZF, Zhuo M (2001). Genetic enhancement of inflammatory pain by forebrain NR2B overexpression. Nat Neurosci.

[B44] Setou M, Nakagawa T, Seog DH, Hirokawa N (2000). Kinesin superfamily motor protein KIF17 and mLin-10 in NMDA receptor-containing vesicle transport. Science.

[B45] Wong RW, Setou M, Teng J, Takei Y, Hirokawa N (2002). Overexpression of motor protein KIF17 enhances spatial and working memory in transgenic mice. Proc Natl Acad Sci USA.

[B46] Guillaud L, Setou M, Hirokawa N (2003). KIF17 dynamics and regulation of NR2B trafficking in hippocampal neurons. J Neurosci.

[B47] Liauw J, Wu LJ, Zhuo M (2005). Calcium-stimulated adenylyl cyclases required for long-term potentiation in the anterior cingulate cortex. J Neurophysiol.

[B48] Toyoda H, Wu LJ, Zhao MG, Xu H, Zhuo M (2007). Time-dependent postsynaptic AMPA GluR1 receptor recruitment in the cingulate synaptic potentiation. Dev Neurobiol.

